# Fatal Pembrolizumab‐Induced Stevens‐Johnson Syndrome/Toxic Epidermal Necrolysis in a Patient With Advanced Lung Adenocarcinoma

**DOI:** 10.1002/kjm2.70132

**Published:** 2025-10-23

**Authors:** Po‐Chih Chang, Ting‐Wei Chang, Chia‐Yu Kuo, Yi‐Zhe Wu

**Affiliations:** ^1^ Division of Thoracic Surgery, Department of Surgery Kaohsiung Medical University Hospital Kaohsiung Taiwan; ^2^ School of Medicine College of Medicine, National Sun Yat‐Sen University Kaohsiung Taiwan; ^3^ Division of Pulmonary and Critical Care Medicine, Department of Internal Medicine Kaohsiung Municipal Siaogang Hospital Kaohsiung Taiwan; ^4^ Department of General Medicine Kaohsiung Medical University Hospital Kaohsiung Taiwan

Dear Editor,

Pembrolizumab, a programmed death‐1 (PD‐1) immune checkpoint inhibitor (ICI), has transformed the management of advanced lung adenocarcinoma [1]; however, immune‐related adverse events (irAEs) such as Stevens‐Johnson syndrome (SJS) and toxic epidermal necrolysis (TEN) can be life‐threatening [2]. Here, we report a fatal case of pembrolizumab‐induced TEN in a 74‐year‐old male with advanced lung adenocarcinoma, highlighting the rapid progression and severe outcomes despite timely medical intervention.

The patient, previously independent in daily activities, was diagnosed with lung adenocarcinoma with bone metastases (cT4N2M1c, stage IVB). Molecular profiling revealed KRAS G12C and TP53 mutations, with programmed death‐ligand 1 (PD‐L1) expression of 15%. His medical history included chronic obstructive pulmonary disease, non‐ST elevation myocardial infarction with prior percutaneous coronary intervention, atrial fibrillation, heart failure with preserved ejection fraction, dyslipidemia, and occult hepatitis B infection.

The patient received the first cycle of chemotherapy with pemetrexed (500 mg/m^2^) and cisplatin (50 mg/m^2^) on March 29, 2024; then, on April 26, he received pembrolizumab (100 mg) in combination with a second cycle of pemetrexed. Ten days later, he developed a generalized skin rash and skin tenderness (Figure 1A). By May 13, he presented with fever and chills. Suspecting SJS, the medical team initiated methylprednisolone and diphenhydramine. Despite aggressive treatment, the patient experienced rapid clinical deterioration, with epidermal detachment involving 65% of the total body surface area (TBSA) (Figure 1B). He was transferred to a burn intensive care unit on May 20 but succumbed to critical against‐advice‐discharge 4 days later.

**FIGURE 1:**
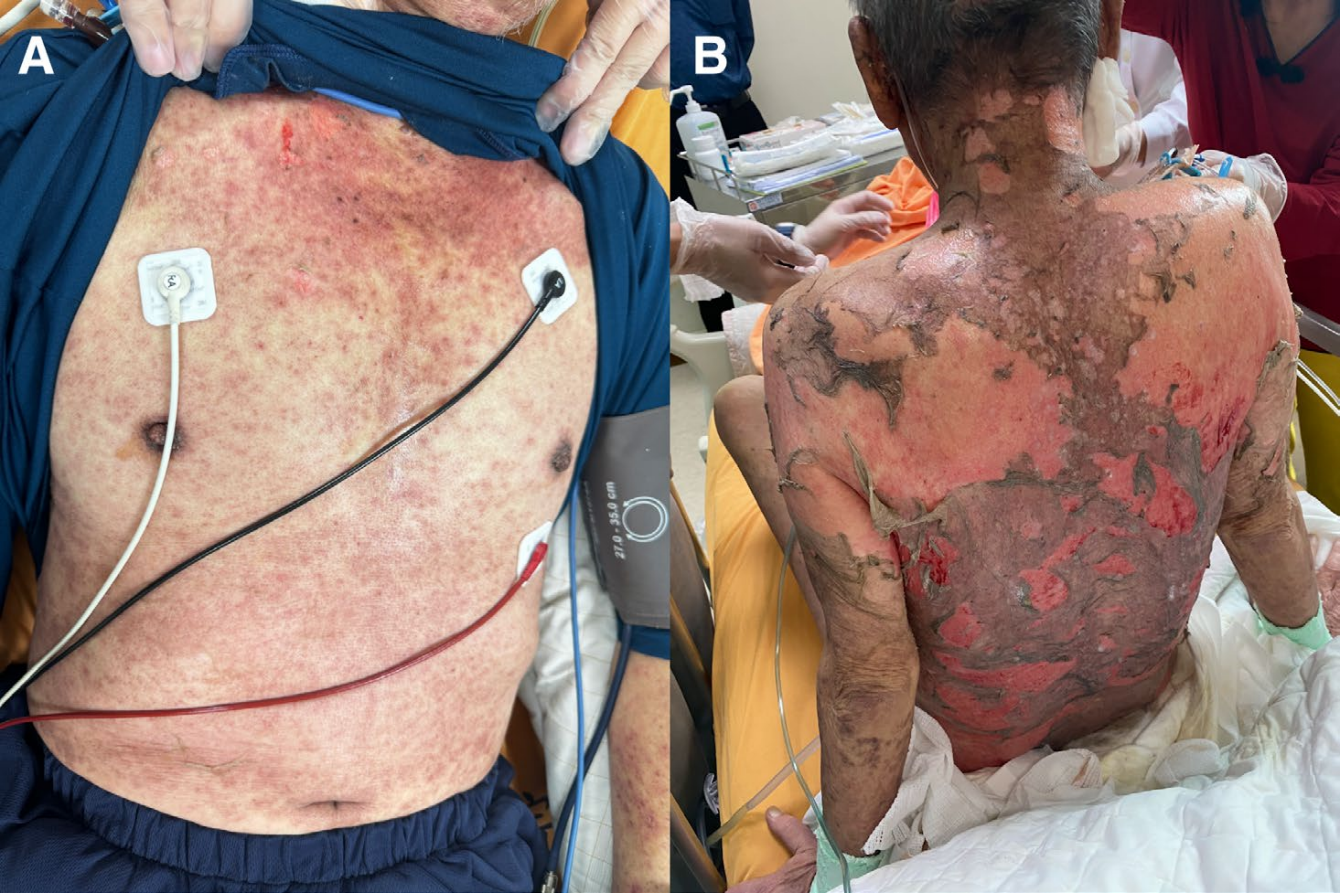
(A) Generalized skin exanthematous rashes involving the trunk on Day 10 after pembrolizumab administration. (B) Progression to toxic epidermal necrolysis (TEN) with 65% total body surface area skin detachment on Day 24.

SJS/TEN secondary to ICIs is a rare but serious irAE. The onset of symptoms in our case aligns with previous reports, where pembrolizumab‐induced SJS/TEN typically manifests within the first two cycles [3]. Extensive TBSA involvement and rapid progression contributed to the fatal outcome. Literature suggests that mortality correlates with higher TBSA involvement (> 30%) and inadequate response to immunosuppressive therapy [3]. Maloney et al. reported a median onset of 11 weeks for pembrolizumab‐induced SJS/TEN [4], emphasizing the need for early detection. Akpala et al. noted that PD‐1 inhibitors, including pembrolizumab, pose a significant risk for SJS/TEN with high morbidity and mortality [5]. Early and aggressive intervention is critical in improving outcomes, although there is no standardized treatment for ICI‐related SJS/TEN, and management relies on clinical experience. Common therapies include systemic corticosteroids, intravenous immunoglobulin (IVIG), cyclosporine, and tumor necrosis factor alpha (TNF‐α) inhibitors, but no treatment has been definitively shown to improve mortality [3]. The current consensus for first‐line treatment of ICI‐related SJS/TEN is systemic corticosteroids (methylprednisolone 1–2 mg/kg/day), with early initiation being essential given the rapid progression and prognostic significance of skin loss. As second‐line options, cyclosporine and TNF‐α inhibitors may help suppress the inflammatory cascade, reduce epidermal necrosis, and promote faster recovery [3]. Plasmapheresis has been rarely reported as a second‐line therapy for ICI‐related SJS/TEN [3]. However, a retrospective cohort study found that it did not reduce either mortality or the length of hospitalization in patients with SJS/TEN.

This case underscores the importance of vigilance in identifying pembrolizumab‐associated SJS/TEN. Clinicians should maintain a low threshold for discontinuation and initiate aggressive management, particularly in elderly patients with comorbidities. Future research is warranted to refine risk stratification and establish optimal treatment strategies for checkpoint inhibitor‐induced SJS/TEN.

## Conflicts of Interest

The authors declare no conflicts of interest.

## Data Availability

The data that support the findings of this study are available from the corresponding author upon reasonable request.
